# Key considerations to improve the normalization, interpretation and reproducibility of morbidity data in mammalian models of viral disease

**DOI:** 10.1242/dmm.050511

**Published:** 2024-03-05

**Authors:** Jessica A. Belser, Troy J. Kieran, Zoë A. Mitchell, Xiangjie Sun, Kristin Mayfield, Terrence M. Tumpey, Jessica R. Spengler, Taronna R. Maines

**Affiliations:** ^1^ Influenza Division, Centers for Disease Control and Prevention, Atlanta, GA 30329, USA; ^2^ Franklin College of Arts and Sciences, University of Georgia, Athens, GA 30602, USA; ^3^ Division of Scientific Resources, Centers for Disease Control and Prevention, Atlanta, GA 30329, USA; ^4^ Division of High-Consequence Pathogens and Pathology, Centers for Disease Control and Prevention, Atlanta, GA 30329, USA

**Keywords:** Ferret, Pathogenesis, Virus

## Abstract

Viral pathogenesis and therapeutic screening studies that utilize small mammalian models rely on the accurate quantification and interpretation of morbidity measurements, such as weight and body temperature, which can vary depending on the model, agent and/or experimental design used. As a result, morbidity-related data are frequently normalized within and across screening studies to aid with their interpretation. However, such data normalization can be performed in a variety of ways, leading to differences in conclusions drawn and making comparisons between studies challenging. Here, we discuss variability in the normalization, interpretation, and presentation of morbidity measurements for four model species frequently used to study a diverse range of human viral pathogens – mice, hamsters, guinea pigs and ferrets. We also analyze findings aggregated from influenza A virus-infected ferrets to contextualize this discussion. We focus on serially collected weight and temperature data to illustrate how the conclusions drawn from this information can vary depending on how raw data are collected, normalized and measured. Taken together, this work supports continued efforts in understanding how normalization affects the interpretation of morbidity data and highlights best practices to improve the interpretation and utility of these findings for extrapolation to public health contexts.

## Teaser

**Summary:** Conscientious analyses of weight loss and temperature readings from mammalian models of viral disease can improve translation of preclinical infection studies for public health research.

## Introduction

Well-characterized small mammalian models contribute invaluable information to studies of human viral pathogens (Ruiz et al., 2013) and provide data that are often included in critical pandemic risk assessment rubrics, in which elevated risk scores indicate viruses that can cause severe and/or fatal disease in relevant mammalian species (Cox et al., 2014; WHO, 2016). Mammalian models are also used pre-clinically to study the effectiveness of therapeutic interventions such as vaccines or antiviral treatments. Diminished clinical signs of infection in these models are often used as key indicators of the therapeutic efficacy of such interventions (Belser et al., 2017; Zivcec et al., 2020). For these reasons, researchers must responsibly interpret data generated from mammalian models to carefully contextualize results with human infection.

Changes in body temperature and loss of body weight post inoculation are two key parameters captured during the acute phase of infection and are often documented in tandem with additional clinical signs (Belser et al., 2016). Daily temperature readings are typically measured using a subcutaneous transponder placed prior to experimentation (Maxwell et al., 2016), and body weight is recorded with a scale. Ideally, both temperature and weight are measured at approximately the same time each day to reduce variation stemming from circadian rhythms or behavioral patterns (Sengupta et al., 2019). Other measures, such as lethargy (Reuman et al., 1989), can contribute valuable information to our understanding of the full spectrum of disease during infection but are inherently more subjective to interpretation by laboratory staff. In contrast, temperature and weight loss are key variables that represent quantifiable, objective datapoints, serving as robust measures that can be performed at different institutions by different personnel.

Nevertheless, in studies of viral pathogenicity in small mammalian models, temperature and weight loss measurements can exhibit a high degree of variability among animals and/or among experiments. This variability can be due to biological and physiological factors associated with animal strains or species, the pathogenesis of the infectious agent, the experimental design (such as the challenge dose and inoculation route) and/or the data collection approaches used, which can be affected by the time that baseline measures are obtained and whether change is calculated as an absolute or percentage change. Although age- and sex-matched inbred strains (such as mice) can provide generally comparable baseline measurements pre-inoculation, outbred models (such as ferrets) can exhibit greater heterogeneity, as we discuss later in this Special Article. Moreover, variability between different strains of the same viral pathogen can lead to divergent presentations of disease post inoculation. For example, ferrets inoculated with influenza A viruses (IAVs) present with a range of pathogenic outcomes during infection, depending on a wide range of factors including, but not limited to, differences in host adaptation, molecular determinants of virulence (which differ between strains) and inoculation protocols (Belser et al., 2016, 2020; Moore et al., 2014). These differences can result in infections that range from asymptomatic to lethal disease. Even when testing the same virus in the same animal species, laboratory-specific and protocol-specific confounders can lead to disparate results between institutions (Belser et al., 2022). This heterogeneity in data and data collection highlights the need to reassess how morbidity measures in virus-infected small mammalian models are recorded, normalized, interpreted and presented, in order to meaningfully extrapolate experimental results to human health and to facilitate the contextualization of results across publications.

**Fig. 1.**
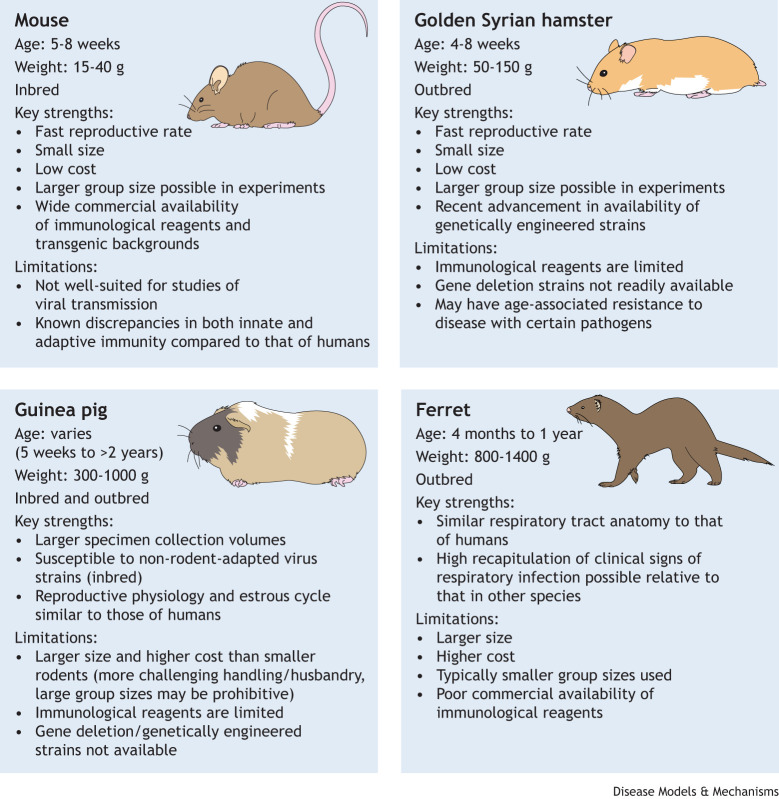
**Comparison of four mammalian models frequently used to study viral pathogens.** Mice, golden Syrian hamsters, guinea pigs and ferrets are used in a range of research and public health applications to study viral pathogens (see Table 1), but possess different key strengths and limitations, including but not limited to those depicted here. The typical age and weight at time of use (infection, vaccination, etc.) are shown for each mammalian model, and for guinea pigs, outbred animals are typically used at younger ages, whereas inbred animals are used at wider age range.

Here, we discuss inherent heterogeneity in temperature and weight loss parameters both within and among study populations, depending on the virus and mammalian species used. We focus on frequently studied viral pathogens that pose a threat to human health and on four small mammalian species commonly used to model viral infection: mice, hamsters, guinea pigs and ferrets (Fig. 1). From an analysis of data aggregated from IAV-infected ferrets as a representative example, we also provide guidance on best practices for the collection and normalization of weight loss and temperature measures collected during experimentation, and the extensive scope of analyses that can be performed on these quantitative data.

**Table 1.**
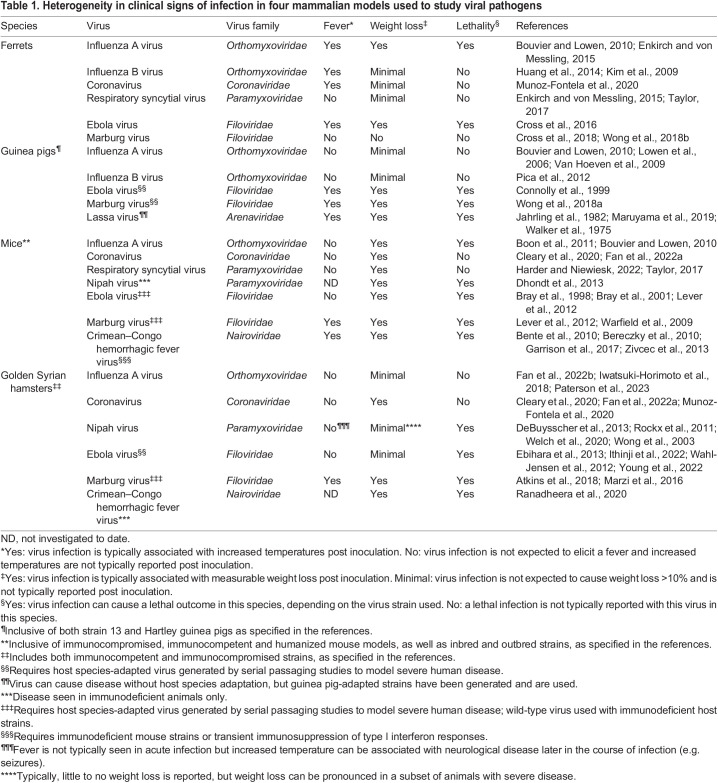
Heterogeneity in clinical signs of infection in four mammalian models used to study viral pathogens

## Heterogeneity among mammalian models of viral pathogenesis

Mice, hamsters, guinea pigs and ferrets are among the small mammalian species most commonly used to research viral pathogens (Ruiz et al., 2013). Table 1 provides an overview of some of the frequently detected signs of morbidity that are present in different species inoculated with viruses relevant to public health, but it does not capture the full range of variability in presentation of clinical signs based on host and virus properties. Following viral infection, the presentation of morbidity-associated clinical signs can vary depending on both the model species and virus characteristics. For example, when mice or ferrets are inoculated with highly pathogenic avian influenza viruses or the reconstructed 1918 pandemic H1N1 virus, they can develop a severe and fatal infection, whereas similarly infected guinea pigs exhibit only a mild and transient illness (Van Hoeven et al., 2009).

As shown in Table 1, the choice of animal model can greatly impact the presence and magnitude of clinical signs that develop post inoculation. The severity of weight loss and survival outcomes differed between mice, hamsters and ferrets following monoinfection with IAV or severe acute respiratory syndrome coronavirus 2 (SARS-CoV-2), or coinfection with both viruses (Wong et al., 2023), demonstrating that differing genetic backgrounds of mammalian species can cause the presentation of clinical signs post inoculation to vary greatly. This requires researchers to select the most appropriate species to study when a specific disease course or clinical outcome is desired (such as lethality or survival). As an example, the infection of over 20 different inbred mouse strains with the same highly pathogenic H5N1 IAV resulted in pathogenic outcomes post inoculation that varied from asymptomatic to fatal disease, depending on the genetic background of the mouse (Boon et al., 2011). These differences can be attributed to both viral load and the varying elicitation of host responses among different strains and species of mice (Boon et al., 2011; Samet and Tompkins, 2017). Furthermore, inbred and outbred strains of the same mammalian species can lead to different pathogenic outcomes post inoculation, as is the case for Lassa virus: the virus is more lethal to inbred strain 13 guinea pigs than to outbred Hartley guinea pigs (Jahrling et al., 1982). SARS-CoV-2 disease severity has also been shown to vary in the hamster model depending on the age, sex and genetic background of the species (Gruber et al., 2022). Similarly, transgenic mice expressing humanized ACE2 (hACE2) receptors display clinical signs similar to those of human COVID-19, whereas in most wild-type mice, SARS-CoV-2 does not robustly replicate (Munoz-Fontela et al., 2020). The magnitude of the clinical signs in these transgenic mice nonetheless varies depending on the particular hACE2-expressing strain used and the age of the animals (Sun et al., 2020). Mouse models that are immunocompromised or deficient for key immune signaling responses (e.g. interferons) frequently show increased morbidity and mortality following viral inoculation relative to those of wild-type strains, as has been shown for Crimean–Congo hemorrhagic fever virus (Bente et al., 2010; Bereczky et al., 2010; Garrison et al., 2017; Zivcec et al., 2013), filoviruses (Bray et al., 1998, 2001; Lever et al., 2012), IAVs (Szretter et al., 2009) and others. Collectively, these studies highlight that close attention must be paid to the influence that the genetic background of a model species might have, following inoculation with a particular viral pathogen, on the scope and severity of clinical signs expected.

Beyond the genetic background of the host, clinical signs associated with morbidity following viral infection can also vary in response to the viral strain and species they are exposed to, and the degree of pathogen–host adaptation that has occurred prior to experimental use. Specifically, when a wild-type virus is not associated with substantial clinical signs in an animal model species, viruses can be adapted via serial passage in the host to confer susceptibility to disease by changing their ability to evade immune defense mechanisms and altered pathogenicity (e.g. replication kinetics and tissue tropism). This results in a model system that more faithfully recapitulates human disease in a desired species. This approach has been used to develop disease models in small animal species that are susceptible to infection by wild-type viruses but that do not develop overt clinical signs post inoculation. Ebolaviruses have been adapted to increase the severity of clinical signs in mice and guinea pigs post inoculation, including weight loss and temperature changes, leading to lethal outcomes. Many human and avian IAVs have also been adapted to elicit weight loss and lethal disease in mouse models. These models have been created to evaluate efficacy of vaccines and therapeutic agents against influenza virus strains that do not cause measurable clinical signs in mice (Choi et al., 2017; Majde et al., 2007). These examples, among others, demonstrate the value of manipulating viral strains to produce measurable signs of morbidity (notably weight loss and lethal outcome) in certain animal models.

Overall, many factors pertaining to both the virus and the model organism must be considered when designing experiments to study viral pathogens in small mammalian models. Public health risk assessment activities often use wild-type viruses and genetically unmodified mammalian hosts. In contrast, in pre-clinical assessments of vaccines or therapeutic agents, a genetically modified virus and/or host model species associated with meaningful, quantifiable differences in morbidity parameters may be chosen to evaluate the efficacy of therapeutics (Zivcec et al., 2020). As typical group sizes used in pathogen infection studies can vary by species (Fig. 1), considerations regarding which species recapitulates experimentally desired clinical signs and provides statistical power to produce robust conclusions are also critical. Regardless of the choice of virus or model species used in infection studies, the collected clinical data must also be accurately quantified and presented.

In the following sections of this Special Article, we focus on temperature and weight data, using aggregated temperature and weight loss data from a representative outbred species (ferrets) inoculated with a representative viral pathogen that can be associated with both mild and severe disease in this species (IAV), to demonstrate conclusions drawn herein.

## The importance of normalizing serially collected temperature and weight data

The ferret model is frequently used to assess the virulence of IAV, but data generated from this model must be carefully interpreted post observation. Box 1 provides details of the previously published, aggregated ferret data used in examples of normalization and analysis approaches discussed in more detail below. These analyses are meant to provide meaningful contextual information to illustrate the need for data normalization and present real-world examples of how the factors discussed can modulate conclusions drawn from data collected for public health risk assessment purposes. From these analyses, we aim to highlight best practices and critical considerations when analyzing data from experiments that capture these parameters.

**Box 1. Use of aggregated ferret data to provide data-driven examples**

**Fig. 2.**
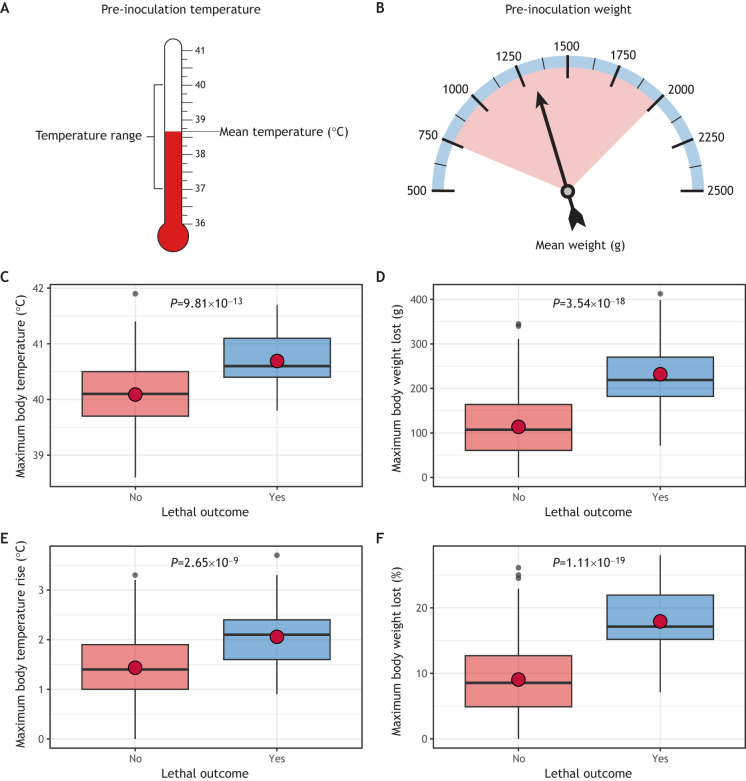
**Variability in non-normalized temperature and weight measurements in ferrets used for influenza A virus risk assessment studies.** (A,B) Prior to inoculation with virus, ferrets can exhibit substantial variability in pre-inoculation temperature and weight. Among ferrets previously used to study virus pathogenicity of influenza A virus (IAV) (see Box 1 for a description of the data used to conduct these analyses), pre-inoculation baseline temperatures ranged from 37 to 40°C, with a mean temperature of 38.7°C (A) and pre-inoculation baseline weights ranged from 764.4 to 2056 g, with a mean weight of 1275.3 g (B). (C,E) Temperature data for IAV-infected ferrets in which the viral infection did or did not produce a lethal outcome during a 14-day post-inoculation (PI) observation period. The highest recorded temperature (non-normalized data) (C) and the maximum increase in body temperature (normalized data) (E) are shown. (D,F) Weight loss data for IAV-infected ferrets in which the viral infection did or did not produce a lethal outcome during a 14-day PI observation period. Maximum body weight loss in grams from the pre-inoculation baseline (non-normalized data) (D) and maximum percentage weight loss from the pre-inoculation baseline (normalized data) (F) are shown. In box-and-whiskers plots, boxes show the interquartile range, the central line marks the median, the red dot depicts the mean, and whiskers show the upper and lower 25% of values. Significance was determined using a two-tailed unpaired Welch's *t*-test. *n*=353 ferrets. See Fig. S1 for a more technical presentation of these analyses. Collectively, these analyses indicate that there is substantial baseline variability in temperature and weight among animals used for risk assessment purposes, with statistically significant differences among peak measurements of both temperature and weight loss independent of data normalization.

**Fig. 3.**
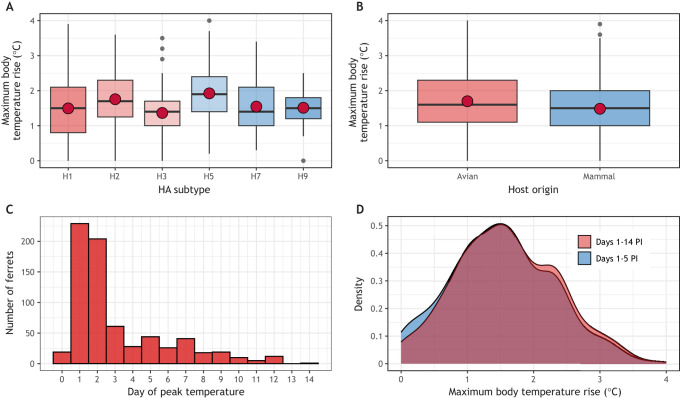
**Normalized temperature increases in ferrets infected with influenza A virus.** (A,B) Among ferrets previously used to study virus pathogenicity of influenza A virus (IAV) (see Box 1 for a description of the data used to conduct these analyses), data were stratified by the hemagglutinin (HA) subtype of viruses used for inoculation (A) or by the host origin of the inoculating virus (B). The plots show that infection with many different viral subtypes (A) and host origins (B) is capable of eliciting elevated peak temperature readings (>1°C) between days 1 and 14 post inoculation (PI) in ferrets. Median (horizontal lines) and mean (red dots) values were generally similar for each set of aggregated data. Boxes depict the interquartile range and whiskers show the upper and lower 25% of values. See Fig. S2 for a more technical presentation of these analyses. (C) Day of peak temperature reading recorded from days 1 to 14 PI. Peak temperature readings in IAV-inoculated ferrets often occur within the first few days post inoculation. (D) Density plot depicting the overlap between maximum body temperature rise recorded during days 1-14 PI (red) or days 1-5 PI (blue). *n*=717 ferrets. Collectively, these analyses indicate that a diversity of IAVs both well and poorly adapted to cause mammalian infection are nonetheless capable of eliciting elevated peak temperature readings in ferrets post inoculation, with potential utility in using different gating strategies to define the time range in which peak measurements are collected and reported.

**Fig. 4.**
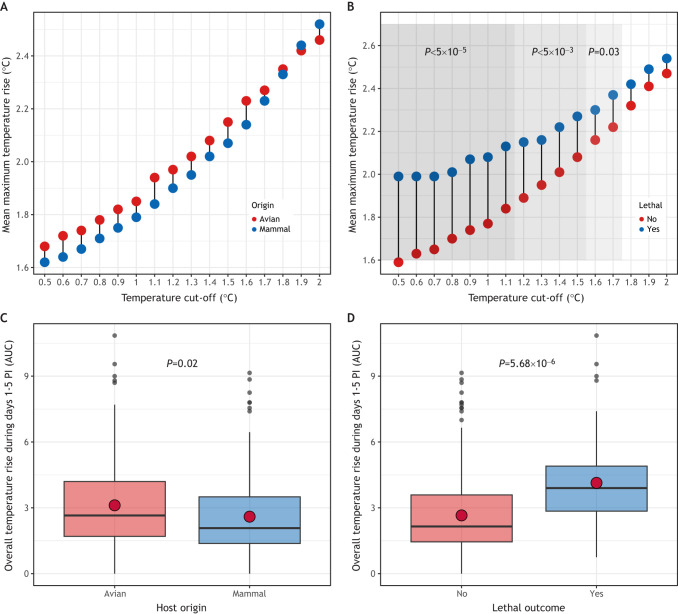
**Analysis of increased body temperature threshold and sustained temperature increases in ferrets infected with influenza A virus.** (A,B) Among ferrets previously used to study virus pathogenicity of influenza A virus (IAV) (see Box 1 for a description of the data used to conduct these analyses), data were stratified by the host origin of the virus (A) or whether the inoculating virus was associated with a lethal outcome (B). The mean temperature increase recorded during days 1-5 post inoculation (PI) was analyzed. The *x*-axis shows the different temperature threshold cut-offs and the *y*-axis shows the mean peak temperature rise (in °C) greater than or equal to the specified cut-off. The shading represents statistical significance, determined using a two-tailed unpaired Welch's *t*-test. *n*=717 ferrets. These graphs show that use of different temperature cut-offs can result in statistically significant differences in mean peak temperature rise across different groups of data, but only when stratified with certain variables (e.g. with lethal outcome but not host origin in the example here). (C,D) Box-and-whiskers plots of the area under the curve (AUC) of temperature increase recorded during days 1-5 PI was analyzed from data stratified by host origin of the virus (C) or whether the inoculating virus was associated with a lethal outcome (D). Boxes show the interquartile range, the central line marks the median, the red dot depicts the mean, and whiskers show the upper and lower 25% of values. Statistical significance was determined using a two-tailed unpaired Welch's *t*-test in C,D. *n*=353 ferrets. These graphs indicate that AUC can represent a meaningful way to quantify elevated temperature in serially collected data, with the degree of statistical significance dependent on the variable for which data are stratified. See Fig. S3 for more a more technical presentation of these analyses.

**Fig. 5.**
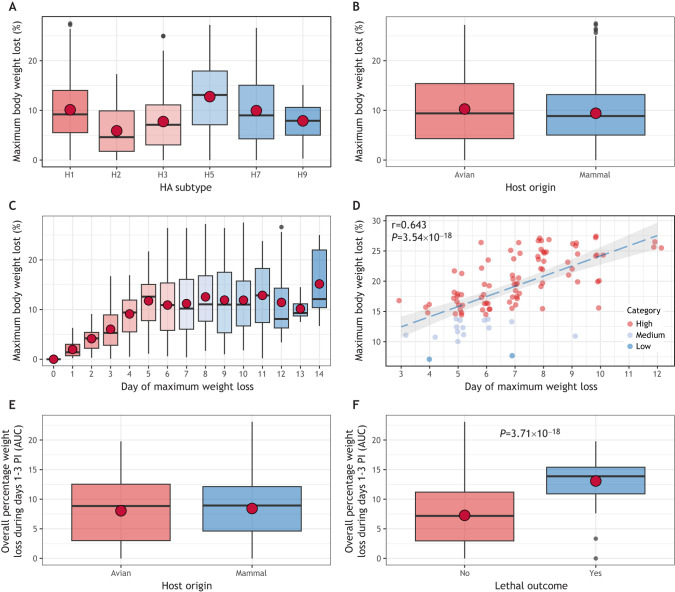
**Normalized weight loss in ferrets infected with influenza A virus.** (A,B) Among ferrets previously used to study virus pathogenicity of influenza A virus (IAV) (see Box 1 for a description of the data used to conduct these analyses), data were stratified by the hemagglutinin (HA) subtype of the virus used for inoculation (A) or by the host origin of the inoculating virus (B). The plots show that infection with many different viral subtypes (A) and host origins (B) can be associated with varying degrees of maximum body weight loss (expressed as a percentage of pre-inoculation body weight) between days 1-14 post inoculation (PI) in ferrets. Median (horizontal lines) and mean (red dots) values were generally similar for each set of aggregated data. Boxes depict the interquartile range and whiskers show the upper and lower 25% of values. (C) The day of maximum weight loss percentage detected occurred between days 1 and 14 PI. (D) Among ferrets with a lethal outcome following IAV infection, there was a statistically significant linear correlation between the day of maximum weight loss observed and the maximum body weight lost. Dots represent individual ferrets that have been categorized into low, medium or high levels of weight loss. (E,F) Box-and-whiskers plots of the area under the curve (AUC) of maximum body weight loss recorded during days 1-3 PI from data stratified by host origin of the virus (E) or whether the inoculating virus was associated with a lethal outcome (F). Statistical significance in F was determined using a two-tailed unpaired Welch's *t*-test in D,F. *n*=717 ferrets (A-D) or 353 ferrets (E,F). These graphs support that AUC can represent a meaningful way to quantify weight loss detection in serially collected data, with the degree of statistical significance dependent on the variable for which data is stratified. See Fig. S4 for more a more technical presentation of these analyses. Collectively, these data indicate that trends in normalized weight loss data can be observed in ferrets post inoculation with a diverse range of IAVs, from both peak recorded values and from serially collected datapoints. However, the way in which data are stratified can reveal trends in data not uniformly present among all variables.

All data-driven analyses presented here were conducted with previously published data from 717 ferrets inoculated with influenza A virus (IAV) by a single research group over ∼20 years (Creager et al., 2023; Kieran et al., 2024). We aim to provide a representative example of how factors discussed throughout this Special Article affect results obtained from studies evaluating viral pathogenicity for public health risk assessment purposes. All ferrets used in these studies were male and between 5 and 12 months of age and serologically negative to currently circulating influenza A and B viruses prior to inoculation. Ferrets were inoculated intranasally (1 ml) with a high dose (10^5^-10^7^ infectious units) of wild-type IAV (inclusive of H1, H2, H3, H5, H7 and H9 subtypes, 125 unique IAVs total), and weight loss and temperature measurements were collected once daily for 14 days post inoculation. All animals were successfully inoculated with each virus, as determined by the presence of infectious virus in serially collected nasal wash specimens. Ferrets that lost >25% of pre-inoculation body weight or exhibited neurological complications were humanely euthanized; lethal events were recorded for both avian- and mammalian-origin viruses and were analyzed on a per-animal basis. All animal work was conducted under the guidance of the Institutional Animal Care and Use Committee at the Centers for Disease Control and Prevention in an Association for Assessment and Accreditation of Laboratory Animal Care (AAALAC) International-accredited animal facility. Experiments were conducted at either biosafety level 2 or 3 containment, including enhancements, as required by the US Department of Agriculture and the Federal Select Agent Program. All analyses presented were conducted in R using version 4.2.1. Figs 2–5 show generalized results; Figs S1–S4 show a more detailed presentation of these analyses.

Ferrets are outbred, and so their baseline temperatures and weights can vary greatly pre-inoculation, even when researchers use age- and sex-matched animals obtained from the same commercial breeding facility (Belser et al., 2022; Stark et al., 2013). Some experimental designs preferentially use animals of the same approximate weight, such as drug delivery studies, in which treatment is administered based on weight, and inhalation delivery methods, in which the respiration of comparable minute volumes of an agent by all animals during a single exposure is required (Gustin et al., 2011; Mrotz et al., 2022). Nevertheless, animals within the same experimental groups frequently exhibit unavoidable levels of variability in baseline parameters. To demonstrate and determine the scope of variability possible in these baseline parameters, we aggregated pre-inoculation weights (in grams) and temperatures (in degrees Celsius) from ferrets used in standard IAV pathotyping studies (see Box 1 for details of the source data). In this aggregated data, ferret pre-inoculation temperatures ranged from 37 to 40°C, and weights ranged from <800 to >2000 g (Fig. 2A,B), supporting baseline variability present in these parameters among animals employed for routine public health risk assessment activities.

Changes in ferret body temperature and weight are typically recorded over a 14-day observation period post inoculation in order to capture clinical signs of infection. However, although absolute changes in these values might be statistically significant (Fig. 2C,E), these data can be challenging to meaningfully interpret when compared to non-normalized baselines. Importantly, the absolute differences in weight or temperature observed in ferrets during an experiment from one institution or research group might not be readily comparable to absolute differences in these parameters reported by another institute and/or research group that might be using animals with different pre-inoculation baseline means. Researchers also set criteria for humane endpoints prior to the start of infection studies, which must then be uniformly applied to animals with differing baseline values. Given the expected variability in baseline parameters, these criteria generally set normalized benchmarks for intervention. For these reasons, reporting percentage weight change over time, calculated by dividing serially collected weight measurements post inoculation by a pre-inoculation baseline, can permit more meaningful analyses than a total measurement of maximum weight in grams (Fig. 2D). Similarly, post-inoculation temperature readings are typically reported as values in degrees Celsius added to or subtracted from pre-inoculation values (Fig. 2F). These normalizations can retain (and even enhance) statistically significant differences present in the data, as trends that are otherwise obscured by the noise of pre-existing variability present in this outbred species might become apparent.

## Analysis and interpretation of increased body temperature post inoculation

As with other viral pathogens in this species (Table 1), ferrets can exhibit elevated body temperatures during acute influenza virus infection, which are typically reported as the mean or median maximum rise in temperature post inoculation. For the aggregated data used here, mean and median values are similar, as depicted in the figures accompanying this Special Article. However, deviations between mean and median values can occur when animals in an experimental group exhibit clinical courses that are not uniform. Here, using aggregated data from ferrets (Fig. 3A), we show a diversity of influenza viruses that cause different median peak elevated temperatures from the baseline. These various IAV subtypes, associated with sustained circulation in humans (e.g. H1 and H3 subtypes) or in zoonotic reservoirs (e.g. H5 and H7 subtypes) can cause median peak elevated temperatures of >1°C post inoculation. These increased temperatures are caused by viruses of either mammalian or avian origin (Fig. 3B), further highlighting the diversity of influenza viruses capable of causing elevated temperatures in animals during the acute phase of infection.

### Considerations for length of observation period

Although normalization to pre-inoculation baselines is a critical step, there are a variety of important factors pertaining to collection and analyses of corresponding data. For example, investigators might have some flexibility in the time period over which to record these measures. Although 14 days is a standard observation period for influenza virus pathotyping studies, virus is typically cleared from the upper respiratory tract of ferrets within the first 7 days post inoculation (PI) (Belser et al., 2022). When ferrets are inoculated with IAV, temperature increases from the pre-inoculation baseline can be measured within 12 h PI (Maines et al., 2012). Their temperatures then typically peak on days 1-3 PI (Fig. 3C), thereafter returning to pre-inoculation levels concurrently with viral clearance. Researchers could, therefore, choose to report temperature changes over a narrower post-inoculation time window that more closely mirrors the period of active virus replication. As an example, we compared the maximum body temperature rises post inoculation detected during a 14- or 5-day observation window in aggregated ferret data. Peak temperature readings recorded during the first 5 days PI generally showed similar trends to those recorded over the 14-day PI observation window (Fig. 3D). These findings highlight the benefits of using gating strategies to more accurately capture maximum temperature increases during periods of active virus replication in animal models.

### Considerations for assessing clinically relevant temperature changes from the baseline

The baseline body temperature of small mammals such as ferrets can vary from day to day in the absence of viral infection. This makes it challenging to determine whether temperature increases recorded post inoculation in this model represent meaningful changes that are due to infection. To account for this, many research groups set a cut-off (e.g. 1°C above baseline) to differentiate between baseline variability and infection-associated increases. However, systematic analyses of variance in mean differences in peak temperature, using different baseline cut-offs, are not frequently conducted. To this end, using aggregated ferret data, we filtered temperature changes using a range of stepwise cut-offs (0.5-2°C) and analyzed peak temperature increases among animals for which a rise in temperature at or above the specified cut-off was reported, with data stratified by host origin of the virus or lethal outcome. As stratification by virus host origin revealed generally comparable peak temperatures (Fig. 3B), unsurprisingly, statistically significant differences were not noted between avian- and mammalian-origin viruses when setting different temperature threshold cut-offs (Fig. 4A). However, when stratifying by lethal outcome (see Box 1 for humane endpoint criteria and how lethal events were analyzed), we found that lower temperature threshold cut-offs were associated with the largest separation in mean peak temperature differences, with separation (and corresponding statistical significance) between groups narrowing as the cut-off value increased (Fig. 4B). These analyses indicate that different gating strategies can be employed depending on the specific experimental groups under investigation, such that relative differences in temperature metrics are most meaningfully interpreted between groups. For example, these analyses support the utility of critically assessing what magnitude of temperature rise is most meaningful for a specific pathogen being studied, or if different temperature cut-offs are necessary when assessing the same viral pathogen across different mammalian species.

### Considerations for assessing and reporting the duration of elevated temperatures

The analyses discussed above rely on recording the peak temperature reading within a defined observation period. However, virus-inoculated ferrets can exhibit elevated temperatures that are sustained over multiple days during the observation period; therefore, reporting peak temperature increase above the baseline cannot capture longitudinal trends. When warranted by the data, researchers might instead elect to graph daily normalized temperature readings to illustrate the differences in this parameter among various experimental groups (Stark et al., 2013; Zitzow et al., 2002). Area under the curve (AUC) measurements can also be used to capture the overall increase above the baseline across successive days (e.g. days 1-5 PI) and can provide a quantitative measure of this event. Analyses of aggregated ferret data revealed comparable AUC values for ferrets inoculated with either avian- or mammalian-origin viruses, in agreement with similar peak mean temperatures for these groups (Fig. 4C). However, in ferrets exhibiting lethal disease, serially collected temperature readings produced significantly higher AUC measurements than those obtained from ferrets that survived the infection, indicating that lethal disease was associated with prolonged detection of elevated temperatures within the first 5 days PI (Fig. 4D).

## Analysis and interpretation of weight loss post inoculation

Weight loss due to inappetence during the acute phase of infection is a common observation in models of severe viral infection and a common presentation of morbidity captured in IAV-inoculated ferrets (see references in Table 1). This parameter is also critical for determining humane experimental endpoints (Hankenson et al., 2013) and many studies use a threshold of a 20-25% reduction in the pre-inoculation body weight of an animal (University of North Carolina at Chapel Hill Standard on Humane Endpoints in Rodents; Talbot et al., 2020). Normalized weight loss data are typically reported by graphing daily weight percentages per ferret or as a group mean, and/or by reporting a peak maximum weight loss, often contextualized with the day post inoculation that the peak was noted. Analyses of aggregated ferret data supports the notion that although various IAV subtypes can cause meaningful weight loss in ferrets, the magnitude of weight loss detected post inoculation can be highly strain dependent, even among genetically related viruses from the same viral subtype (Fig. 5A). Furthermore, weight loss post inoculation can be detected at similar dynamic ranges after infection with an IAV of mammalian or avian origin (Fig. 5B), supporting weight loss as a critical parameter to capture in all experiments.

### Considerations for assessing clinically relevant weight loss

Similar to temperature, ferrets exhibit daily fluctuations in weight independent of viral infection, making it a challenge to ascertain what percentage of weight loss from the pre-inoculation baseline represents a meaningful change that is due to infection. In this model, weight loss fluctuations <5% of pre-inoculation baseline are typically attributed to normal variation and not to viral infection, although this can depend on the animals and viral strain used. Weight loss >5% of the pre-inoculation baseline typically falls outside of this normal fluctuation and is often presented as a mean of the maximum weight loss percentage per virus. Analyses of expected variation independent of infection are important to perform for other species to aid in differentiation of notable weight variation from acceptable variation based on species, strain and age used. To further investigate the utility of weight loss data, we explored associations between maximum weight loss and disease severity. Weight loss data aggregated from ferrets observed for 14 days following inoculation with IAV were grouped into quartile categories (none, low, medium or high). Firstly, this analysis showed that ferrets typically entered the higher weight loss category at later time points post inoculation, relative to infection with viruses that elicited a lower overall weight loss (Fig. 5C; Fig. S4C). Similarly, ferrets with a lethal outcome exhibited a linear correlation between time and maximum weight loss; ferrets that survived for a longer period lost more weight before succumbing to IAV infection than ferrets that were humanely euthanized earlier during the observation period (Fig. 5D).

### Considerations for assessing and reporting duration of weight loss

Weight loss during a 14-day observation period can be mild and transient or can be sustained for several days, with or without returning to pre-inoculation baselines. As such, although the reporting of maximum weight loss can indicate the relative severity of this parameter, it does not capture the duration of weight loss observed over multiple days. Reporting the length and corresponding magnitude of weight loss could aid in comparisons of pathogenicity and therapeutic efficacy. AUC measurements can be used to assess the rapidity of observed weight loss and the persistence of sub-baseline weight to differentiate between transient and resolving changes and weight loss that is sustained for prolonged periods of time. In analyses of aggregated ferret weight data collected days 1-3 PI, AUC values were generally comparable in ferrets inoculated with either avian- or mammalian-origin viruses, in agreement with similar peak mean weight loss detected between these groups (Fig. 5E). However, we observed significantly higher AUC measurements during this same time window in ferrets with a lethal outcome, relative to measurements of ferrets that survived the infection, indicating that lethal disease is associated with a more rapid and pronounced weight loss in the first 3 days PI (Fig. 5F). Although our analyses with AUC were limited to days 1-3 PI, we found in our aggregated data that mean weight following IAV infection in ferrets continued to decrease until day 5 PI, and the day of maximum weight loss could be detected between days 1 and 14 PI (Fig. 5C). This suggests that the degree of weight loss relative to time might be a useful parameter to measure (notably in the context of assessing endpoint criteria), although this might be subject to several host-specific and virus-specific features that fall outside the scope of what is presented here.

## Conclusions

As shown in Table 1, recording daily perturbations in the body temperature and weight of small mammalian models following viral infection is a nearly ubiquitous component of the experimental protocols that govern modeling of disease *in vivo*. The analyses we present here show that the conclusions drawn from these data can vary depending on the way in which raw data are normalized, gated and measured. We also highlight here the advantages and limitations of different analysis strategies, depending on their specific application and on how this information is interpreted in the context of different experimental studies (Table 2). A better understanding of the complex virus–host interactions that govern the clinical signs we seek to measure and interpret in infection studies can also inform the choice of the most appropriate animal model to use for a given experimental study. This becomes particularly important when using results obtained from *in vivo* infection studies to inform decisions concerning human public health.

**Table 2.**
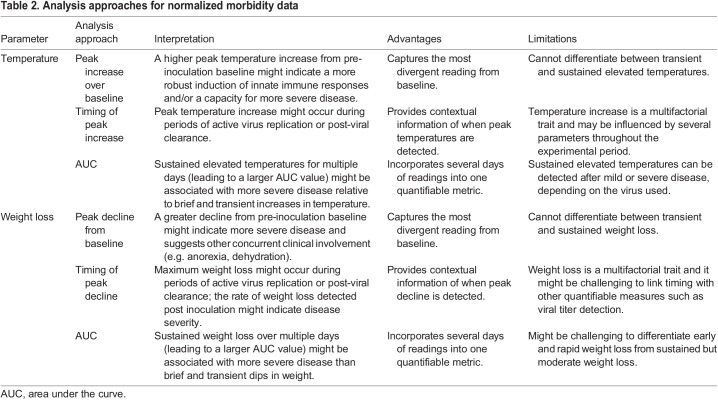
Analysis approaches for normalized morbidity data

The established models and examples discussed in this Special Article do not represent a complete list of all parameters that might modulate disease severity post viral infection. It would be wise to examine normalization approaches for other factors not specifically examined here, including sex, age and pre-existing immunity. Sex effects are important to consider because, depending on the viral pathogen investigated, differences in sex effects within a species might be apparent, specifically related to factors such as weight, for which normal growth curves are known to differ between sexes (Francis et al., 2021; Sabikunnahar et al., 2022). To take the sex effects on weight into account, experimental designs might choose to perform combined and separate analyses of results from both sexes. As mentioned above, the age of animals being studied can also result in differing levels of pathogenicity detected (Bissel et al., 2019; Francis et al., 2021; Martins et al., 2022). In addition, studies in small mammalian models typically use animals that are serologically naïve to the pathogen being investigated. However, it might be desirable to elicit pre-existing immunity to a pathogen (by means of prior infection and/or vaccination) to assess the capability of novel and emerging viruses to cause meaningful disease in the presence of more complex immunological backgrounds, to better emulate immune responses in human populations (Music et al., 2019). As inclusion of these and other related variables to study designs might introduce additional heterogeneity, considering best normalization approaches prior to analyses with these data will be critical.

The analyses we present here of data collected from IAV-infected ferrets are intended to provide examples of how these values can be normalized and interpreted; they are not intended to represent the only approaches that might be suitable for collecting and/or analyzing quantifiable morbidity data. Although it is standard practice in the field to collect morbidity measurements at approximately the same time each day, with many studies employing a subcutaneous transponder for temperature measurements (as was the case with the ferret data described in Box 1), other experimental designs using different protocols to collect these data (e.g. use of continuous body temperature telemetry or other collection modalities) may warrant different interpretation approaches (Maxwell et al., 2016; Mei et al., 2018; Roberts et al., 2012). Furthermore, a detailed examination of the underlying causes of morbidity measurements presented in Table 1 lie outside the scope of this article. Elevated temperatures and/or reductions in body weight are often associated with increased inflammatory processes and/or anorexia (Ruiz et al., 2013), but can also be caused by primary effects of viral replication and/or post-viral clearance owing to secondary complications once the acute infection itself has resolved. Similarly, as supported by Fig. 5D, lethality outcomes following viral challenge can occur throughout the observational period post inoculation, during periods of active virus replication (attributable to cytokine storms, systemic spread of virus, etc.), and during post-viral clearance (attributable to persisting tissue damage or secondary infections, development of neurological signs due to virus invasion to the central nervous system, etc.) (see references in Table 1). The choice of virus strain, host species and genetic background, and protocol-specific parameters can influence the timing and magnitude of all of the morbidity parameters discussed here, underscoring the need to pay close attention to these factors prior to commencing experiments, to ensure that the collection and analyses of morbidity measurements are appropriate for the study design and research outcomes.

Although the clinical signs in this article are limited to temperature, weight loss and lethal outcomes, the collection of additional morbidity parameters might also be appropriate and warrant a consideration of the most appropriate ways by which to normalize and analyze these observations. For example, although lethargy assessments were not included here due to potential observational bias, the development of video tracking technology can overcome limitations of manual scoring of such signs to provide quantifiable measurements of this parameter (Oh et al., 2015). Ultimately, understanding not just the need to capture morbidity data but also the most rigorous way to report and analyze this information ensures that the results obtained are of the highest applicability to the field. Such rigor in approach will contribute to necessary efforts of model refinement and best practices in animal research (Balls and Straughan, 1996).

## Supplementary Material

10.1242/dmm.050511_sup1 Supplementary information
