# Supplementary material for: Key considerations to improve the normalization, interpretation and reproducibility of morbidity data in mammalian models of viral disease
Source: Dis Model Mech. 2024 Mar 5;17(3):dmm050511. doi: 10.1242/dmm.050511 (PMC10941659; doi:10.1242/dmm.050511)
Supplement: Supplementary information [file dmm-17-050511-s1.pdf]

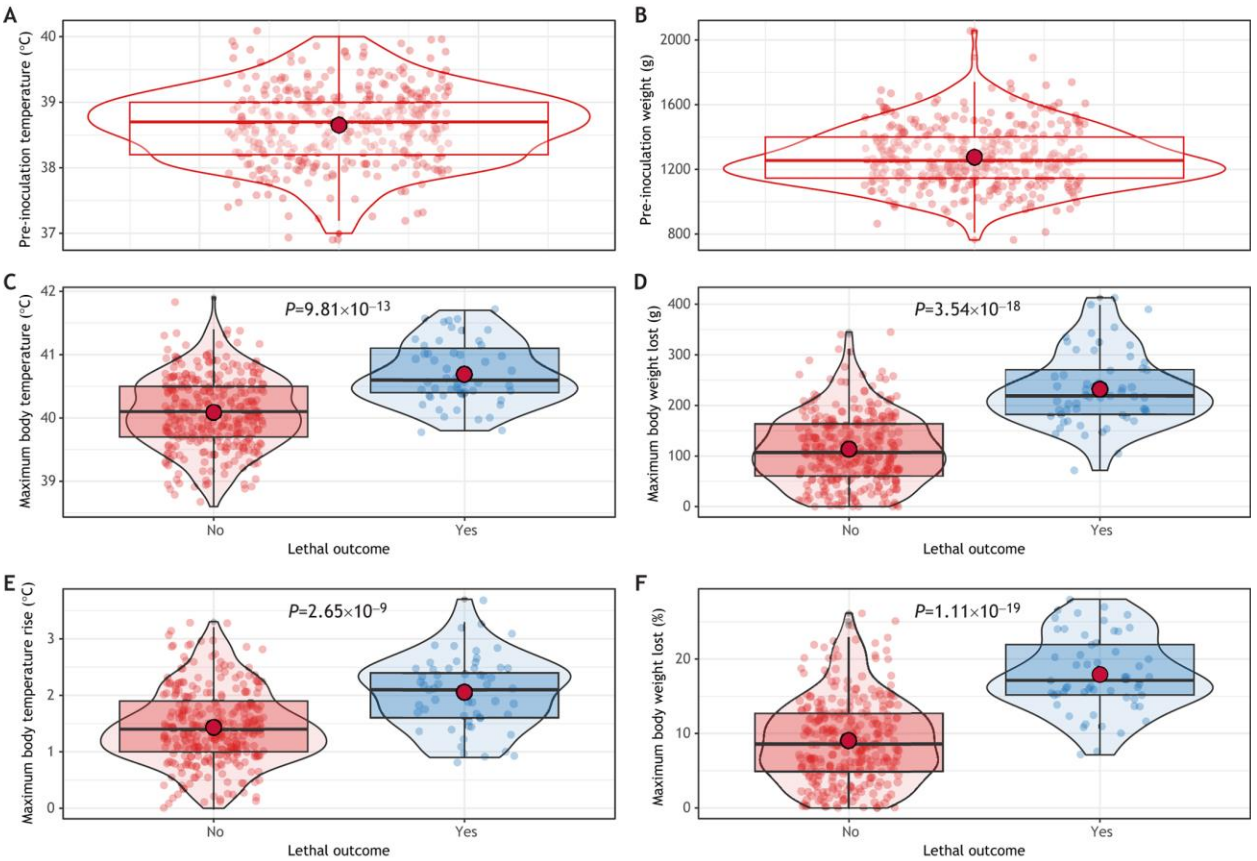

Fig. S1. Variability in non-normalized temperature and weight measurements in ferrets. (A,B) Pre-inoculation temperature (°C) (A) and weight (g) (B) of individual ferrets. (C,D) Maximum body weight loss (g) from the pre-inoculation baseline (non-normalized data, C) or maximum percentage weight loss from the pre-inoculation baseline (normalized data, D) among ferrets that did or did not exhibit a lethal outcome during a 14-day post-inoculation observation period. (E,F) Highest recorded temperature (°C) (non-normalized data, E) or maximum increase in body temperature (°C) (normalized data, F) among ferrets that did or did not exhibit a lethal outcome during a 14-day post-inoculation observation period. In box-and-whiskers plots, boxes show the interquartile range, the central line marks the median, the red dot depicts the mean, and whiskers show the upper and lower 25% of values. Significance was determined using a two-tailed unpaired Welch's *t*-test (using the ggbetweenstats package in R). *n*=353 ferrets.

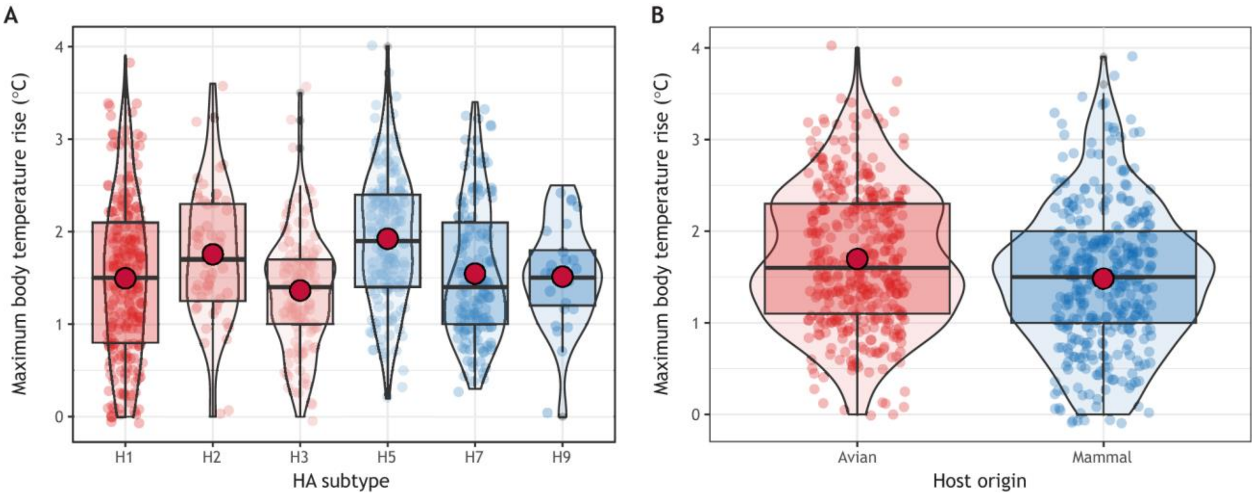

Fig. S2. Normalized temperature increases in ferrets post inoculation with influenza A virus. (A,B) Maximum rise in body temperature (°C) among ferrets inoculated with influenza A virus (IAV) of different hemagglutinin (HA) subtypes (A) or host origin (B). In box-and-whiskers plots, boxes show the interquartile range, the central line marks the median, the red dot depicts the mean, and whiskers show the upper and lower 25% of values. *n*=717 ferrets.

Disease Models & Mechanisms • Supplementary information

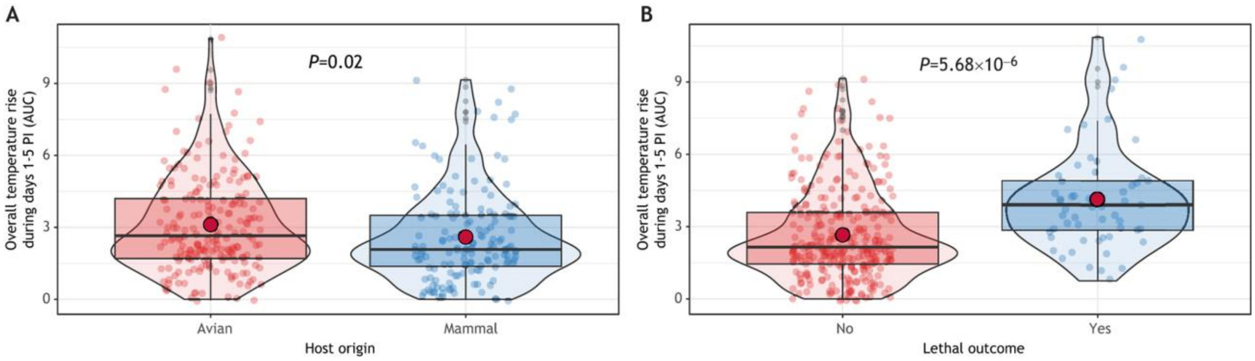

Fig. S3. Analysis of increased body temperature threshold and sustained temperature increases in ferrets post inoculation with IAV. (A,B) Box-and-whiskers plots of area under the curve (AUC) of temperature increases during days 1-5 PI (n=353 ferrets), stratified by either host origin (A) or lethal outcome (B). Boxes show the interquartile range, the central line marks the median, the red dot depicts the mean, and whiskers show the upper and lower 25% of values. Statistical significance was determined using a two-tailed unpaired Welch's t-test.

Disease Models & Mechanisms • Supplementary information

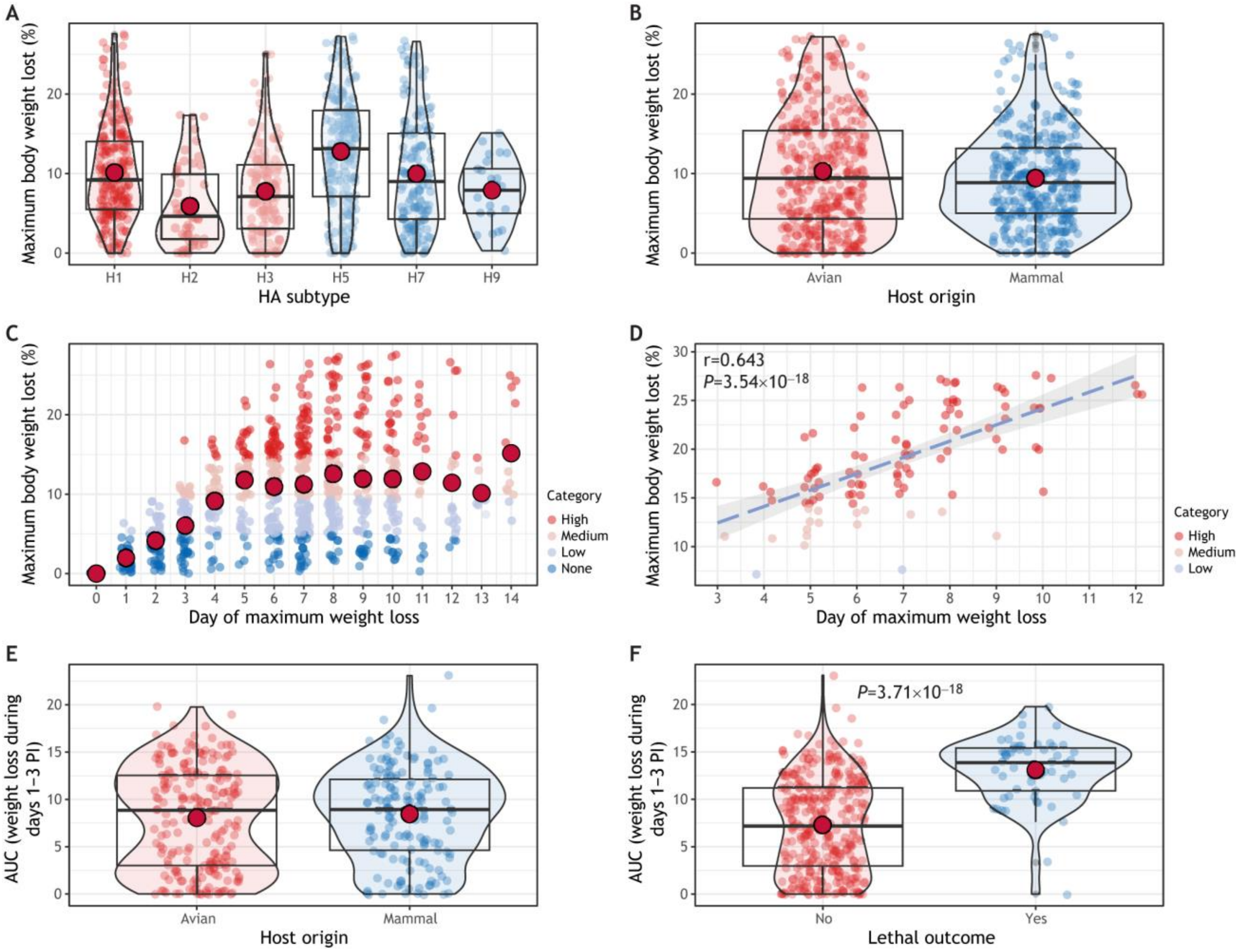

Fig. S4. Normalized weight loss in ferrets post inoculation with IAV. (A,B) Maximum body weight loss (expressed as a percentage of the pre-inoculation baseline weight) among ferrets inoculated with IAV of different subtypes (A) or host origin (B). (C,D) Day post inoculation when maximum body weight loss was observed among all ferrets (C) or ferrets with a lethal outcome only (D). Weight loss categories: none (<5%), low (5-9.5%), medium (9.5-14.5%) and high (14.5-27.5%). Red dots depict the mean per day. Pearson correlation coefficient as the line with standard error is shown; the *P*-value represents Pearson product-moment correlation. (E,F) Box-and-whiskers plots of area under the curve of weight loss during days 1-3 PI, stratified by either viral host origin (E) or lethal outcome (F). Statistical significance in F was determined using a two-tailed unpaired Welch's *t*-test. In A,B,E,F, boxes show the interquartile range, the central line marks the median, the red dot depicts the mean, and whiskers show the upper and lower 25% of values. *n*=717 ferrets (A-D) or 353 ferrets (E,F).
